# Heterologous Expression and Enzymatic Properties of β-Glucuronidase from *Clostridium perfringens* and Its Application in Bilirubin Transformation

**DOI:** 10.3390/microorganisms13051043

**Published:** 2025-04-30

**Authors:** Qianlin Wu, Qing Guo, Fo Yang, Mengru Li, Yumeng Zhu, Binpeng Xu, Lu Zhao, Shanshan Zhang, Youyu Xie, Feng Li, Xiaomin Wu, Dayong Xu

**Affiliations:** 1 Anhui Province Key Laboratory of Pollutant Sensitive Materials and Environmental Remediation, Huaibei Normal University, Huaibei 235000, China; 17334612971@163.com (Q.W.); yangfo0820@163.com (F.Y.); 18256188142@163.com (M.L.); 12211070750@chnu.edu.cn (Y.Z.); zss9042@163.com (S.Z.); xieyy@chnu.edu.cn (Y.X.); lifeng@chnu.edu.cn (F.L.); 2 School of Life Sciences, Huaibei Normal University, Huaibei 235000, China; 3 Anhui Chem-Bright Bioengineering Co., Ltd., Huaibei 235000, China; liangsui1980@163.com (Q.G.); xvb1996@sohu.com (B.X.); zhaolu@ahkebao.com (L.Z.); 4 Anhui Province Engineering Technology Research Center for Livestock by-Product Medical Intermediate Extraction, Huaibei 235000, China

**Keywords:** β-glucuronidase CpGUS, bilirubin, molecular dynamics simulation, catalytic mechanism

## Abstract

β-glucuronidase is an important hydrolase, which plays an important role in drug metabolism, clinical diagnostics, and biotransformation. This study focuses on the heterologous expression, isolation, purification, and its enzymatic properties of β-glucuronidase CpGUS from *Clostridium perfringens*, as well as its application in the whole-cell transformation of unconjugated bilirubin from pig bile. A recombinant *E. coli* BL21(DE3)/pET-28a-CpGUS was constructed for the heterologous expression of CpGUS, with the majority of the expressed enzyme being soluble. Enzymatic analysis showed that CpGUS displayed optimal activity at pH 5.0 and 45 °C, and it rapidly lost activity at pH < 4.5. Metal ions, such as Mg^2+^ and Fe^2+^, enhanced CpGUS catalysis, while Zn^2+^, K^+^, Fe^3+^, Mn^2+^, Cu^2+^, and Na^+^ inhibited it. Notably, Cu^2+^ and Fe^3+^ can significantly inhibit β-glucuronidase, resulting in the complete loss of its activity. The results of the whole-cell transformation experiment show that when *E.coli* BL21(DE3)/ pET-28a-CpGUS at an OD_600_ of 10 was incubated at pH 5.0, a temperature of 45 °C, and a rotation speed of 200 rpm for 12 h, the hydrolysis rate of the conjugated bilirubin in pig bile reached 81.1%, the yield of unconjugated bilirubin was 76.8%, and the purity of unconjugated bilirubin was 98.2%. The three-dimensional structure of CpGUS was predicted using AlphaFold2 (AlphaFold v2.0, DeepMind Technologise Limited, London, UK), and p-Nitrophenyl-β-D-Glucuronide (pNPG) and conjugated bilirubin were then docked to the CpGUS protein model using SWISSDOCK. The best docked conformations of the CpGUS–pNPG and CpGUS–conjugated bilirubin complex systems were simulated by independent 500 ns molecular dynamics (MD) runs with the RSFF2C force field, and the binding dynamic and catalytic mechanism of each system were obtained. The results indicated that π-π stacking, hydrogen bonding, and hydrophobic interactions between the key residue Tyr472 and the benzene ring of pNPG molecules are crucial for its catalytic process. Similarly, for the binding and catalysis of conjugated bilirubin by CpGUS, the π-π stacking and hydrogen bonding and hydrophobic interactions between the sidechains of residues Phe368 and Tyr472 and the benzene ring of conjugated bilirubin play a synergistic role during its catalytic process. Their total binding free energy (∆*G_bind_*) values were calculated to be as high as −65.05 ± 12.66 and −86.70 ± 17.18 kJ/mol, respectively. These results suggest that CpGUS possesses high binding and catalytic hydrolysis properties for both pNPG and conjugated bilirubin.

## 1. Introduction

β-Glucuronidase (GUS) is a glycosylated hydrolase capable of hydrolyzing β-glucuronic acid (GlcA) residues from glycosides [1]. It cleaves the glucoside bond of β-glucuronic acid at the non-reducing end, releasing glucuronic acid and the associated ligand [2], found across a range of organisms, including bacteria, fungi, plants, and animals. Most are categorized under the Glycoside Hydrolase Family 2 (GH2) [3], while others fall within the GH1 and GH79 families, grouped under GH-A. GUS has been extensively applied in various fields, such as disease regulation [4], drug testing and analysis [5], as reporter genes [6], and in modifying natural products [7]. Recently, GUS has gained significant attention for its role in enhancing the solubility, function, and bioavailability of natural compounds, like triterpenes, flavonoids, alkaloids, and steroids, by removing glycosylated components. Notably, GUS from diverse sources AtGUS from *Aspergillus terreus* Li-20, SpGUS from *Staphylococcus pasteuri* 3I10, and CgGUS from *Chaetomium globosum* has been utilized to convert glycyrrhizic acid to glycyrrhetinic acid [8]. Additionally, LdGUS from *Lactobacillus delbrueckii* Rh2 is employed in the biotransformation of baicalin and Han baicalin [9]. More recently, GUS from *Paenibacillus polymyxa* [10] and StGUS from *Staphylococcus* sp. RLH1 have been used for bilirubin conversion [11].

Bilirubin, a critical lipid-based drug and a major component of various Chinese patent medicine formulations, experiences high market demand and pricing. However, current production methods fall short of meeting this demand due to raw material shortages and low production efficiency. Moreover, bilirubin, a key constituent of the valuable Chinese herbal medicine bezoar, is mainly sourced from animal bile, such as pig bile, where over 90% exists as conjugated bilirubin bonded with one or two β-glucuronic acid molecules, rendering it valueless. The conventional bilirubin production process, involving high temperatures and strong alkalis to remove glucuronic acid groups, tends to oxidize bilirubin to biliverdin, resulting in reduced yield and significant environmental pollution [12]. In recent years, an increasing number of studies have shown that bilirubin is not only an important metabolite in the body but also a potent lipophilic antioxidant. Bilirubin can effectively scavenge various reactive oxygen species (ROS) in the body [13], thereby reducing the damage to cells and tissues caused by oxidative stress. Its unique antioxidant properties endow it with significant biological effects in aspects, such as anti-cancer [14], protection against ischemia-reperfusion injury [15], anti-oxidation, and anti-inflammation [16]. In addition to these important functions of anti-oxidation, anti-inflammation, and protection against ischemia-reperfusion injury, positive research results have also been achieved regarding the application of bilirubin in the treatment of diabetes [17], cardiovascular diseases [18], liver diseases [19], and other conditions. For example, bilirubin has demonstrated certain therapeutic effects in regulating blood glucose levels, improving insulin sensitivity, protecting the cardiovascular system from oxidative damage, and alleviating liver inflammation. These findings have laid the foundation for bilirubin to be considered as a potential drug for the treatment of metabolic diseases.

Consequently, enzymes, serving as principal catalysts in biological transformations, offer unparalleled advantages over chemical synthesis in several respects. Through various bioinformatics approaches, the relationships between enzyme structures and their functions can be elucidated, further allowing for the improvement of their properties [20,21,22]. For example, Weng examined the structure–activity relationship between intestinal bacterial β-glucuronidase and human carboxylesterase 2 flavonoid inhibitors, characterizing the inhibition kinetics and constructing a docking model to determine the molecular binding mechanisms, thus aiding in the optimization of inhibitor designs [23]. In contrast, Wallace BD et al. demonstrated the role of β-glucuronidase in drug metabolism by integrating molecular dynamics simulation and experimental validation, highlighting the importance of key residues, like Glu413, in proton transfer [24]. These studies not only enrich bioinformatics databases but also enhance the optimization of algorithms and models.

In this study, the β-glucuronidase CpGUS gene [25] was obtained from the genome of *Clostridium perfringens* (GenBank: CP076004.1), with its codons optimized for expression in *E. coli* BL21(DE3). The enzymatic properties of purified β-glucuronidase CpGUS were analyzed, and an efficient, environmentally friendly method for producing unconjugated bilirubin from pig bile via whole-cell transformation was explored.

## 2. Materials and Methods

### 2.1. Plasmid and Strain

The codon-optimized β-glucuronidase CpGUS gene was synthesized by Sangon Biotech (Shanghai, China) Co., LTD. This gene was subsequently ligated into the pET28a(+) plasmid vector at the *BamH*I and *Xho*I cloning sites. The recombinant plasmid pET28a-CpGUS was then transformed into *E. coli* BL21(DE3) recipient cells.

### 2.2. Medium

LB medium (g/L): yeast extract 5, sodium chloride 10, tryptone 10, agar 20.

M9 Modified fermentation medium (g/L): disodium hydrogen phosphate 17, potassium dihydrogen phosphate 3, glucose 10, yeast extract 5, ammonium chloride 1, sodium chloride 0.5, magnesium sulfate heptahydrate 0.5, calcium chloride 0.011. This medium was supplemented with 10 mL (1% *v*/*v*) trace element solution per liter.

Trace element solution (g/L): ethylenediamine tetraacetic acid 0.84, cobalt chloride hexahydrate 0.25, manganese chloride tetrahydrate 1.50, copper chloride 0.15, boric acid 0.30, sodium molybdate dihydrate 0.25, zinc acetate dihydrate 1.30, ferric citrate 1.00, soluble in 0.10 mol/L HCL. Bacterial removal was achieved using a 0.22 μm filter membrane.

Supplement medium (g/L): glucose 500, yeast powder 100, peptone 10, magnesium sulfate heptahydrate 7.

50 mM acetic acid-sodium acetate buffer (pH 4, 4.5, 5, 5.5, 6): 0.05 mol/L sodium acetate buffer was prepared, its pH was adjusted with acetic acid and stored at 4 °C for later use.

50 mM Tris-HCL buffer (pH 6.5, 7, 7.5, 8, 8.5, 9, 9.5, 10): Prepare 0.05 mol/L Tris buffer, adjust its pH with HCl and NaOH, and store at 4 °C for later use.

### 2.3. Expression and Purification of CpGUS Protein

Initially, the CpGUS base sequence was cloned into the pET28a(+) expression plasmid, with positive clones screened for kanamycin (Kan) resistance [26] and verified by enzyme digestion. The recombinant plasmid was confirmed by sequencing before transforming into *E. coli* BL21(DE3) recipient cells to obtain the recombinant strain *E. coli* BL21(DE3)/pET28a- CpGUS. This strain was cultured on an LB plate containing kanamycin and incubated overnight at 37 °C. Monoclonal strains were then selected and inoculated into 5 mL of LB medium with kanamycin for overnight culture. Subsequently, 500 mL of the same medium was inoculated with a 1% culture volume and incubated at 37 °C and 220 rpm until the OD_600_ reached 0.6–0.8. IPTG was added, and the culture was maintained at 16 °C and 220 rpm for 16–18 h. Cells were then centrifuged at 8000 rpm and 4 °C for 10 min, and the pellet was stored at −80 °C for later use.

For cell lysis, wet-weighted bacteria were resuspended in lysis buffer (20 mM Tris-HCl pH 8.0, 500 mM NaCl, 5 mM imidazole) at a ratio of 1 g: 10 mL. The mixture was homogenized by vortexing until no visible clumps remained and then disrupted using an ultrasonic disruptor. The lysate was centrifuged at 8000 rpm and 4 °C for 10 min, and the supernatant was collected as the crude enzyme solution. The β-glucuronidase CpGUS, tagged with a 6×His at the N-terminus, was purified using Ni-NTA affinity chromatography [27]. The column was equilibrated with a buffer (20 mM Tris-HCl pH 8.0, 500 mM NaCl, 5 mM imidazole), and the crude enzyme was filtered through a 0.22 μm filter before loading onto the column. After loading, the column was washed with a buffer containing 100 mM imidazole to remove impurities. Elution was performed using a step gradient of imidazole (150 mM to 500 mM), and the eluates were collected [28]. The purified enzyme was then dialyzed overnight in buffer 1 (20 mM Tris-HCl pH 8.0, 500 mM NaCl, 1 mM DTT) followed by buffer 2 (20 mM Tris-HCl pH 8.0, 150 mM NaCl, 1 mM DTT) for 6–8 h. The final product was verified by SDS-PAGE.

### 2.4. Parameter Optimization of CpGUS Fermentation Process

To optimize the soluble expression and activity of CpGUS, the fermentation parameters were adjusted. The impact of various IPTG concentrations (0.005 mM to 0.5 mM) on cell growth and enzyme production was assessed. Cultures were incubated at 37 °C and 220 rpm until the OD_600_ reached 0.6–0.8 and then induced with IPTG at 16 °C for 16–18 h. The induction temperature is critical for promoting proper protein folding and avoiding aggregation into inclusion bodies [29]. The effects of eight different concentrations of the inducer (0.005 mM, 0.01 mM, 0.05 mM, 0.1 mM, 0.2 mM, 0.3 mM, 0.4 mM, and 0.5 mM) on enzyme production were examined. The cultures were incubated at 37 °C and 220 rpm until the OD_600_ reached 0.6–0.8. Following the addition of the inducer, the cultures were induced at 16 °C and 220 rpm for 16–18 h.

Moreover, the induction temperature is a critical factor for the soluble expression of foreign proteins in *E. coli*. Typically, increasing the induction temperature can accelerate protein synthesis. However, if the synthesized protein precursors are not properly folded and transported in a timely manner, they are prone to aggregate into inclusion bodies within the cells. Reducing the induction temperature can improve the soluble expression of these proteins [30]. We investigated the effects of various induction temperatures (16 °C, 20 °C, 25 °C, 30 °C, and 37 °C) on cell growth and enzyme production. Cultures were incubated at 37 °C and 220 rpm until the OD_600_ reached 0.6–0.8. Post-induction, temperatures were adjusted to desired levels, and cultures were induced for 16–18 h at 220 rpm.

In addition, after activation, strains were inoculated in LB liquid medium with kanamycin and incubated at 37 °C until the OD_600_ reached 0.6–0.8. Following the addition of the inducer, the temperature was lowered to optimize enzyme expression. We monitored changes in cell biomass over time to assess the influence of induction time on bacterial growth.

### 2.5. Enzymatic Properties of CpGUS

CpGUS hydrolyzes the substrate 4-nitrophenyl-β-D-glucuronide (pNPG) to produce p-nitrophenol. We determined CpGUS enzymatic activity by hydrolyzing pNPG and used a standard curve of p-nitrophenol at different concentrations in a 50 mM acetoacetic acid-sodium acetate buffer for quantification. Absorbance was measured at 405 nm, and enzymatic activity was calculated accordingly [28].

Using pNPG as the substrate, we determined the optimum pH for CpGUS activity at 40 °C. We pipetted 10 μL of enzyme solution into 40 μL of buffer containing 1.25 mM pNPG at varying pH values. After a 10-min reaction, we added 200 μL of 0.4 M Na_2_CO_3_ to stop the reaction [31]. For pH stability, we repeated this method, incubating the enzyme solution on ice for 30 min, then measuring residual activity at the optimum pH.

We then established a temperature gradient to find the optimal reaction temperature for CpGUS. We pipetted 10 μL of enzyme solution into 40 μL of buffer at an optimal pH and incubated the mixture at various temperatures for 10 min. To assess thermal stability, we incubated the enzyme solution at these temperatures for 30 min, cooled it on ice, and measured residual activity.

We also prepared ionic solutions at specific concentrations, mixed them with the enzyme solution, and incubated the mixture on ice for 30 min before measuring residual enzyme activity at optimal pH and temperature [32]. Control tests used inactivated enzyme solution, and three replicates were conducted.

### 2.6. Determination of Enzyme Kinetic Parameters

Using pNPG as the substrate, we prepared pNPG solutions at concentrations ranging from 0.4 mM to 1.4 mM in 50 mM acetic acid-sodium acetate buffer at pH 5.0. We added 10 μL of enzyme solution to 40 μL of buffer and reacted it at 40 °C for 10 min before quantifying p-nitrophenol. We used the Lineweaver–Burk plotting method to calculate enzyme kinetic parameters [33].

### 2.7. Catalytic Mechanism of CpGUS Hydrolysis of pNPG and Conjugated Bilirubin

As no CpGUS protein crystal structure was available in the PDB database, we predicted its structure using AlphaFold2 [34]. We processed the CpGUS amino acid sequence with the run_docker.py script run by python3 and obtained the 3D model of CpGUS from AlphaFold2. We then conducted molecular docking of pNPG and conjugated bilirubin with the CpGUS protein system using the SWISSDOCK platform (https://www.swissdock.ch accessed on 29 September 2024). After selecting the best-binding conformations, we performed independent MD simulations to determine the binding stability, kinetic characteristics, and thermodynamic properties of the interactions, thus elucidating their catalytic mechanisms; our MD parameters are consistent with our previous systems [35,36].

### 2.8. E. coli BL21(DE3)/pET28a-CpGUS in a 5 L Fermenter

First, *E. coli* BL21(DE3)/pET28a-CpGUS was retrieved from a −80 °C freezer and streaked on a solid LB plate for activation. Subsequently, a single colony was inoculated into LB medium containing kanamycin (50 µg/mL) for overnight culture at 37 °C. The culture was then transferred at a 1% (*v*/*v*) inoculation rate into LB medium with the same kanamycin concentration for an additional 5 h of growth. The culture was added to 3 L of enhanced fermentation medium containing kanamycin M9, with an initial rotation speed of 300 rpm, a pH of 7.0, and a ventilation rate of 3 L/min. As the dissolved oxygen levels decreased, the rotation speed was adjusted to maintain approximately 30% oxygen. Once residual sugars were depleted and oxygen levels recovered, the feed medium was used to adjust the pH back to 7.0 with ammonia water. Upon reaching an OD_600_ of about 20, all residual sugars were consumed. At an OD_600_ of 40, the temperature was reduced to 20 °C. IPTG was then added to a final concentration of 0.5 mmol/L, and fermentation was terminated after 12 h. The endpoint OD_600_ of the fermentation broth was 97.5. The fermentation solution was centrifuged at 6000 rpm at a low temperature for 30 min, the supernatant was discarded, and the bacteria were washed twice with PBS phosphate buffer (pH 7.0) prior to whole-cell transformation.

### 2.9. Whole-Cell Transformation of Pig Bile to Prepare Unconjugated Bilirubin

Initially, pig bile was filtered, and the filtrate was adjusted to pH 5.0 with acetic acid, adding final concentrations of 0.3% sodium bisulfite and 0.05% anhydrous magnesium sulfate. The washed *E. coli* BL21(DE3)/pET28a-CpGUS from high-density fermentation was then added. The OD_600_ of the whole-cell bacteria in the transformation system was set to 10. The hydrolysis reaction proceeded at pH 5.0, 45 °C, and 200 rpm for 12 h. The total bilirubin content at the start and end of the reaction was measured by the diazo method, and the hydrolysis conversion rate of conjugated bilirubin was calculated [37]. This process was repeated three times.

Subsequently, the pH of the hydrolyzed and transformed bile was adjusted to 6.0, and 50% chloroform (*v*/*v*) was added. The mixture was stirred for 3 min, allowed to stand for 10 min, stirred again for 3 min, and then allowed to settle for 30 min. The lower layer was separated using a separatory funnel, repeating the process twice. The separated layer was then transferred to a rotary evaporation flask for evaporation (ensuring the temperature did not exceed 85 °C). After evaporation, bilirubin was dissolved with boiling anhydrous ethanol, filtered under suction, dried, and weighed [37]. The purity of the bilirubin was subsequently determined by HPLC [18]; the compounds were monitored at UV wavelengths of 450nm with acetonitrile−1% glacial acetic acid solution (95:5) as the mobile phase.

## 3. Results

### 3.1. Construction and Expression of Recombinant Plasmid

To characterize the physicochemical properties of CpGUS, the codon-optimized DNA sequence was ligated into the *BamH*I and *Xho*I sites of pET28a(+) to construct the plasmid pET28a(+)-CpGUS. This plasmid was then transformed into *E. coli* BL21 (DE3) for protein expression and purification.

**Figure 1:**
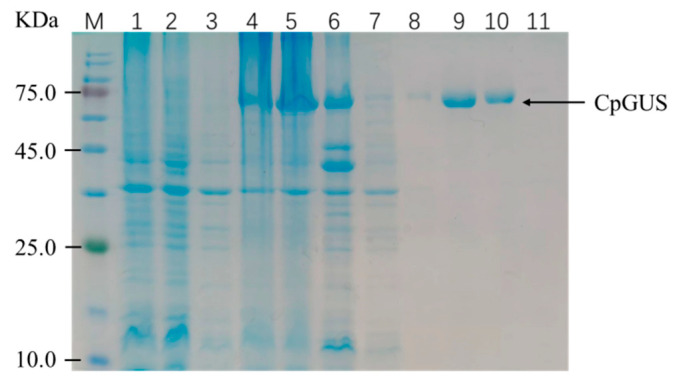
SDS-PAGE analysis of β-glucuronidase CpGUS. M: Protein Marker; 1: no-load crushing total; 2: no-load crushing supernatant; 3: no-load crushing precipitation; 4: CpGUS crushing total; 5: CpGUS crushing supernatant; 6: CpGUS broken precipitation; 7: flow through fluid; 8: 100 mM imidazole elution; 9: 150 mM imidazole elution; 10: 200 mM imidazole elution; 11: 500 mM imidazole elution.

SDS-PAGE analysis revealed that CpGUS was highly expressed in the *E. coli* BL21 (DE3) host. Most of the CpGUS was soluble, eluting at an imidazole concentration of 150 mM. The molecular weight of β-glucuronidase CpGUS was estimated to be approximately 70 KDa based on comparisons with protein marker bands (Figure 1).

### 3.2. Parameter Optimization of Fermentation Process

**Figure 2:**
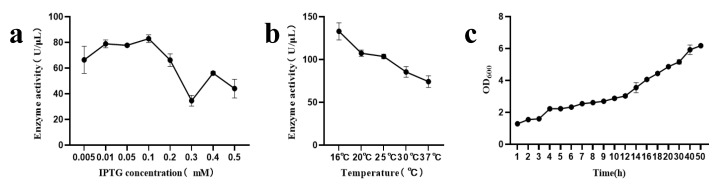
Parameter optimization of β-glucuronidase CpGUS fermentation process. (**a**) Effect of inducer concentration on CpGUS enzyme activity; (**b**) Effect of induction temperature on CpGUS enzyme activity; (**c**) Biomass growth curve over time after adding inducers.

Figure 2a demonstrates that different concentrations of IPTG significantly influenced the soluble expression of CpGUS. Notably, as the inducer concentration increased from 0.05 mM to 0.1 mM, enzyme activity also increased, peaking at 89.2 U/μL at 0.1 mM. However, further increases in inducer concentration led to a decrease in activity; at 0.3 mM, activity dropped to 34.5 U/μL, only 38.7% of the peak value. Figure 2b reveals that of the five temperature gradients tested, the highest enzyme activity (132.9 U/μL) occurred at 16 °C, with the lowest at 37 °C. As the temperature increased, enzyme activity decreased, leading to the selection of 16 °C as the optimal induction temperature. The progression of cell biomass over time after adding inducers is depicted in Figure 2c. During the first 20 h, cell biomass increased steadily, but after 20 h, the rate of increase slowed. Consequently, an induction time of 18–20 h was selected as the optimal point for terminating fermentation in subsequent experiments.

### 3.3. Characteristics of CpGUS

**Figure 3:**
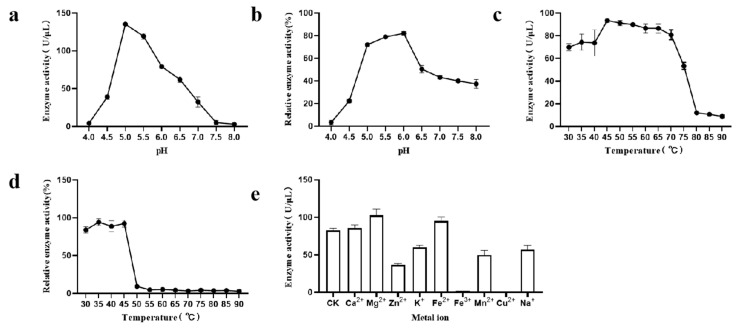
Enzymatic properties of β-glucuronidase CpGUS. (**a**) Optimum reaction pH; (**b**) pH stability; (**c**) Optimum reaction temperature; (**d**) Thermal stability; (**e**) Effect of metal ions on activity.

The optimum pH for CpGUS was 5.0, where its activity peaks at 135.1 U/μL, as shown in Figure 3a. Enzyme activity increased between pH 4 and 5 and declines between pH 5.5 and 8.0. Figure 3b illustrates that CpGUS lost activity under strongly acidic conditions and maintained high residual activity between pH 5.0 and 6.0 but showed reduced activity from pH 6.5 to 8.0. Temperature also markedly affected CpGUS’s activity and stability. As demonstrated in Figure 3c, the optimal temperature for CpGUS was 45 °C, where enzyme activity reached 93.4 U/μL. Figure 3d indicates that CpGUS was thermally stable between 30 °C and 45 °C, maintaining high activity over prolonged periods. However, at 50 °C and above, enzyme activity significantly decreased or disappeared, indicating low heat resistance. Metal ions impacted CpGUS’s catalytic activity variably. Figure 3e shows that Fe^2+^ and Mg^2+^ enhanced CpGUS activity, while Ca^2+^ had a negligible effect. Conversely, Zn^2+^, K^+^, Fe^3+^, Mn^2+^, Cu^2+^, and Na^+^ inhibited its activity, with Cu^2+^ and Fe^3+^ causing complete loss of activity.

### 3.4. Analysis of Kinetic Parameters of Enzymes

**Figure 4:**
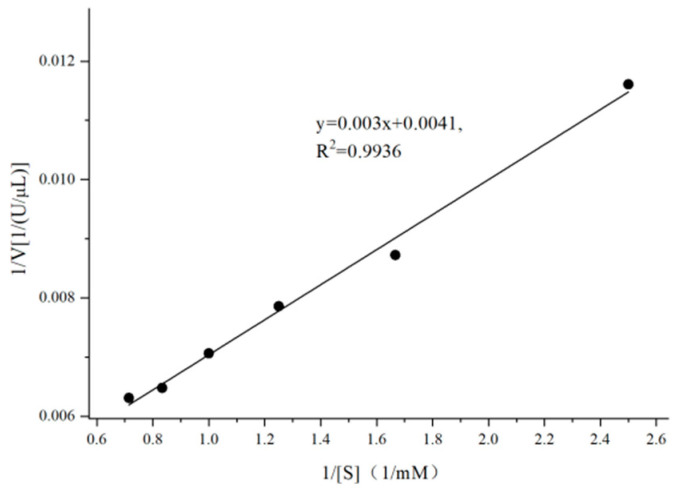
CpGUS kinetics equation.

Enzyme kinetic parameters for CpGUS were determined using double-reciprocal plotting. The results are presented below (Table 1):

As illustrated in Figure 4, the kinetic parameters for CpGUS were: Vmax = 243.90 U/μL, Km = 0.73 mM.

### 3.5. Catalytic Mechanism of CpGUS Hydrolysis of pNPG

**Figure 5:**
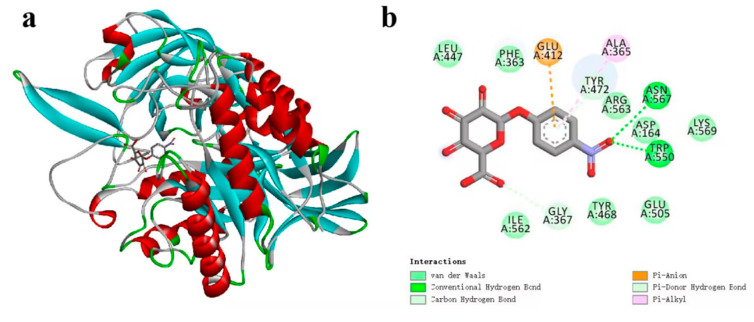
Docked pocket (**a**) and binding mode (**b**) of CpGUS and pNPG.

Figure 5a illustrates the docking pocket and binding modes of CpGUS with pNPG molecules, while Figure 5b shows that pNPG molecules were located within the binding cavities formed by the residues Asp164, Phe363, Ala365, Gly367, Glu412, Leu447, Tyr468, Tyr472, Glu505, Trp550, Ile562, and Arg563.

Molecular docking results suggest that CpGUS binds to pNPG molecules and could be tightly anchored, catalyzing their decomposition with high binding affinity. However, the docked results only partially revealed the static characteristics of enzymatic interactions and lacked detailed insights into the binding stability of the CpGUS–pNPG complex and its catalytic kinetic behavior. To further explore the dynamic equilibrium of this interaction and to enhance enzymatic efficiency, an independent 500 ns MD simulation was conducted with the RSFF2C force field.

**Figure 6:**
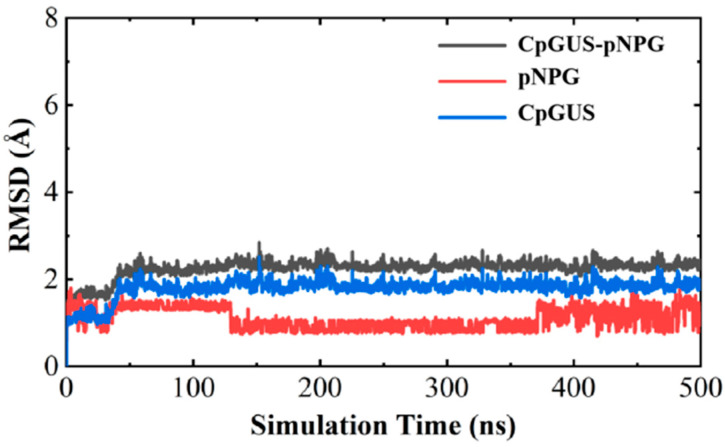
Root mean squared deviation (RMSD) curve of the CpGUS–pNPG complex system as a function of MD simulation time.

The root mean squared deviation (RMSD) curves for the CpGUS–pNPG complex are shown in Figure 6. After initial 30 ns fluctuations, the curves began to rise slightly and stabilized at 50 ns, with its RMSD value of approximately 2.2 Å. This stabilization indicates that the complex system could reach equilibrium within our MD timescale. Furthermore, the RMSD value for the ligand of pNPG in the system decreased from 1.5 to 1.0 Å at 125 ns, remained stable until 375 ns, and then rose again to 1.5 Å. The RMSD value was stabilized below 1.5 Å throughout the MD simulation, suggesting that this system could be tightly bounded and reach equilibrium. This equilibrium of MD trajectory provides a foundation for subsequent calculations of thermodynamic properties of the complex.

The equilibrated process of the CpGUS–pNPG complex was further analyzed to determine the thermodynamic properties of the enzymatic catalytic process. Using the MM/PBSA method, the binding free energy (∆*G_bind_)* of the system was calculated from the equilibrated 300–350 ns MD trajectory. The energy contributions of four free energy components, including van der Waals interactions (Δ*G_vdw_*), electrostatic interactions (Δ*Gele)*, polar solvation free energy (Δ*G_PB_*), and non-polar solvation (Δ*G_SA_*), are listed in Table 2. It can be seen that the ∆*G_bind_* value of the CpGUS–pNPG complex was −65.05 ± 12.66 kJ/mol. The Δ*G_vdw_* and Δ*G_ele_* values were calculated as high as −147.17 ± 14.36 and −119.21 ± 9.53 kJ/mol, respectively, while the Δ*G_SA_* value was relatively low, at −15.30 ± 0.87 kJ/mol. The Δ*G_PB_* value was found to be 216.63 ± 10.94 kJ/mol. When it was at 430–500 ns, the value of ∆*G_bind_* was −61.28 ± 18.01 kJ/mol. The calculated values of Δ*G_vdw_* and Δ*G_ele_* were −127.56 ± 14.62 kJ/mol and −113.37 ± 18.78 kJ/mol, respectively, the value of Δ*G_SA_* was −14.72 ± 0.93 kJ/mol, and the calculated result of the free energy value of Δ*G_PB_* for the complex was 194.37 ± 20.65 kJ/mol. These results imply the significant roles van der Waals and electrostatic interactions, as well as non-polar solvation, play in the catalytic process of the CpGUS–pNPG system, whereas polar solvation interactions impose negatively impacts on its catalytic process. These findings also indicate strong binding interactions between CpGUS and pNPG facilitate their enzyme-substrate recognition.

**Figure 7:**
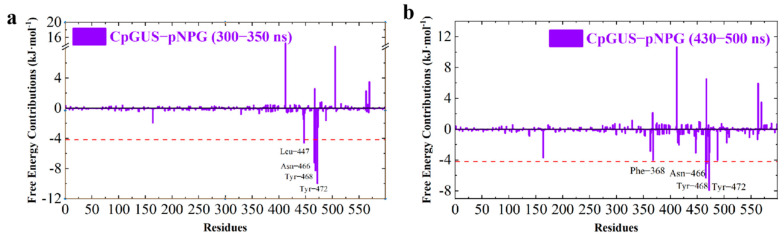
Binding free energy contribution of each residue in the CpGUS–pNPG complex obtained from the equilibrated 300–350 ns (**a**) and 430–500 ns (**b**) MD trajectory.

To explore the key residues involved in its enzyme-substrate recognition and catalytic dynamics, per-residue free energy decomposition (PFED) calculations were performed, and their results are displayed in Figure 7 and listed in Table 3. During the simulation time period of 300–350 ns, four key residues, such as Leu447, Asn466, Tyr468, and Tyr472, significantly contribute to the binding and catalytic interactions, with their corresponding values of −4.60, −7.21, −8.29, and −9.99 kJ/mol. During the simulation time period of 430–500 ns, the residues with a binding free energy contribution value exceeding −4.18 kJ/mol are Asn466, Tyr468, and Tyr472. Their ∆G*_bind_* values are −6.32, −4.47, and −7.92 kJ/mol. These PFED findings further highlight that van der Waals and electrostatic interactions formed between these residues and pNPG make great contributions to their binding recognition and enzyme–substrate catalysis.

**Figure 8:**
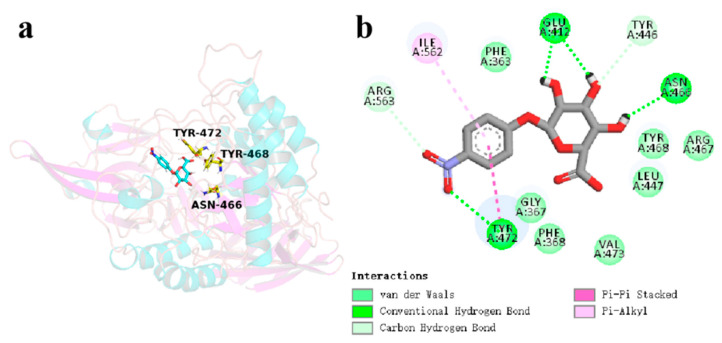
Key interaction analysis of the CpGUS–pNPG system. (**a**) The dominant conformation of CpGUS–pNPG system with key interactions at the active catalytic pocket under the equilibrated state; (**b**) Interaction model diagram of the CpGUS–pNPG system.

As illustrated in Figure 8, the pNPG molecules could be stably bound to the active cavities constituted by several non-polar residues, including Phe363, Gly367, Tyr446, Met448, Leu447, and polar residues, such as Asp164, Glu412, Asn466, Tyr472, Glu505, Phe368, and Tyr468. In addition to the π-π stacking and hydrogen bonding between residues Asn466, Tyr468, and Tyr472 in the CpGUS structure and the benzene ring of pNPG, strong hydrophobic interactions also contribute a lot to the catalytic process.

**Figure 9:**
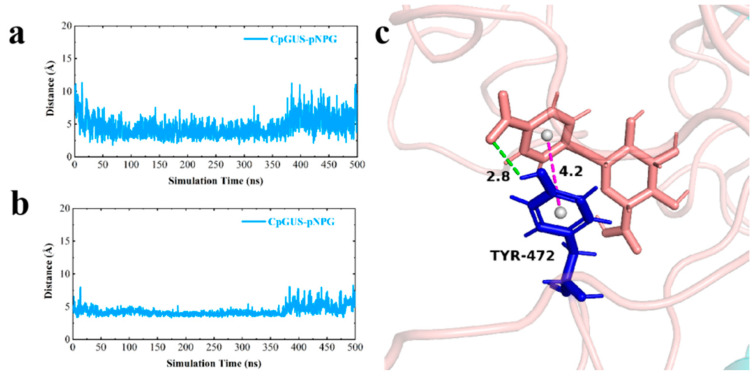
The distance changes between key residues and pNPG as a function of MD simulation time. (**a**) The time-dependent distance changes of the hydrogen bond formed between the key residue Tyr472 and pNPG; (**b**) The time-dependent distance changes of the π-π stacking interaction formed between the key residue Tyr472 and pNPG; (**c**) The ribbon diagram of the distances of the interactions between key residue Tyr472 and pNPG (unit: Å).

Although the structure of pNPG was locally shifted in the CpGUS cavity during the MD equilibrium process, the π-π stacking and hydrogen bonding interactions formed between the sidechain of Tyr472 and the benzene ring of pNPG remained stable in the dominant conformation (Figure 9a,b). These interactions significantly contributed to the binding stability, enabling the pNPG molecules to be tightly anchored at the active catalytic pocket of CpGUS. The hydrogen bond between Tyr472 and pNPG was 2.8 Å, and the benzene ring and pNPG was 4.2 Å (Figure 9c).

### 3.6. Preparation of Unconjugated Bilirubin Through High-Density Fermentation and Whole-Cell Transformation of Pig Bile

**Figure 10:**
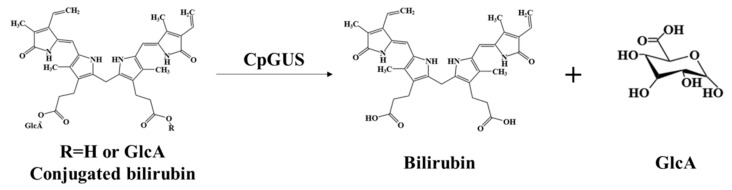
Schematic diagram of β-glucuronidase CpGUS catalyzing the conversion of conjugated bilirubin.

In pig bile, bilirubin is found as conjugated bilirubin. The enzyme β-glucuronidase CpGUS catalyzes the conversion of conjugated bilirubin into unconjugated bilirubin and glucuronic acid, as depicted in Figure 10.

High cell concentrations, achieved through high-density fermentation, were utilized in the conversion process. The methods described in Section 2.8 were employed, achieving an optical density at 600 nm (OD_600_) of 97.5 in the final fermentation broth.

**Figure 11:**
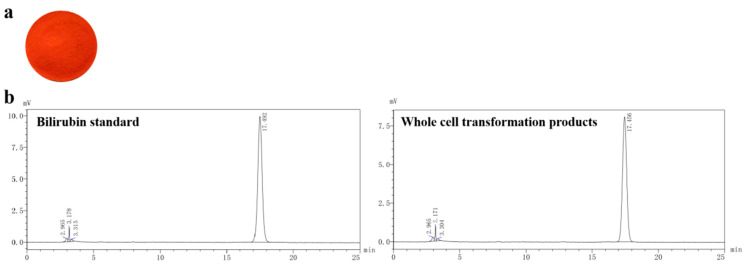
Results of whole-cell transformation test of pig bile. (**a**) Bilirubin extracted by chloroform following hydrolysis and conversion; (**b**) High-performance liquid chromatography of the transformation product alongside a bilirubin standard.

According to Table 4, under these conditions, the hydrolysis rate of conjugated bilirubin was 81.1%. Following hydrolysis, 0.353 g of bilirubin was extracted per liter of pig bile using chloroform, with a yield of 76.8%. The purity of the bilirubin, analyzed by high-performance liquid chromatography (HPLC), was 98.2%, as shown in Figure 11b.

As indicated in the HPLC chromatogram in Figure 11b, the unconjugated bilirubin, produced by the hydrolysis and extraction processes catalyzed by CpGUS, matched the chromatogram of the bilirubin standard. This outcome confirms the efficacy of CpGUS in hydrolyzing conjugated bilirubin in pig bile to yield unconjugated bilirubin.

### 3.7. Catalytic Mechanism of CpGUS Hydrolyzed Conjugated Bilirubin

**Figure 12:**
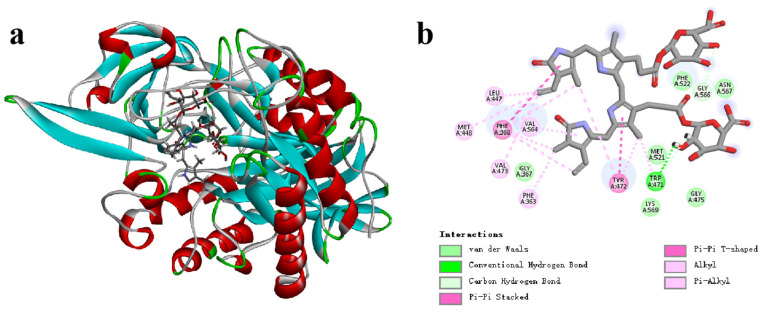
Docked cavity (**a**) and binding mode (**b**) of CpGUS and conjugated bilirubin.

The docking pocket and binding mode of CpGUS with conjugated bilirubin is displayed in Figure 12a. It demonstrates that conjugated bilirubin was also docked within the active cavity of CpGUS. This catalytic cavity was composed of several residues: Phe363, Gly367, Phe368, Leu447, Met448, Trp471, Tyr472, Val473, Gly475, Met521, Phe522, Val564, Gly566, Asn567, and Lys569 (Figure 12b).

**Figure 13:**
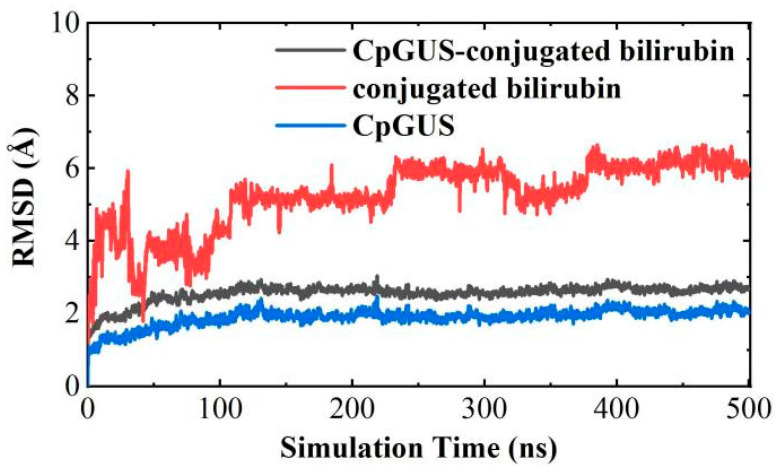
The time-dependent root mean squared deviation (RMSD) curve of CpGUS and the conjugated bilirubin complexed system.

To further investigate the mechanism of β-glucuronidase, CpGUS catalyzed the hydrolysis of conjugated bilirubin, a 500 ns MD run was also simulated and the details about the catalytic interactions of the CpGUS–conjugated bilirubin complex during the equilibrium process were obtained. As shown in Figure 13, the RMSD curve of conjugated bilirubin initially fluctuated at approximately 4.9 Å within several nanoseconds, reached a maximum at 6.0 Å, and then dropped sharply to 2.0 Å. Its RMSD curve increased gradually to 5.2 Å from 120–500 ns, and it fluctuated slightly and stabilized between 5.2 Å and 6.2 Å. However, the RMSD curves of CpGUS and the complex initially increased to 2.0 and 2.8 Å, respectively, and then stabilized during the subsequent MD simulation.

The binding free energy (∆*G_bind_*) of the CpGUS–conjugated bilirubin complex was calculated from different MD equilibrated stage and is listed in Table 5. The ∆*G_bind_* value of the complex increased from −67.97 ± 36.72 to −86.70 ± 17.18 kJ/mol from 150–200 ns to 400–500 ns. During these two equilibrated stages, van der Waals (Δ*G_vdw_*), electrostatic interaction (Δ*G_ele_*), and non-polar solvation free energy items (Δ*G_SA_*) always played dominant roles and significantly contributed to the binding affinity, facilitating the catalytic process of the CpGUS–conjugated bilirubin complex. These findings imply that CpGUS is capable of catalyzing the hydrolysis of conjugated bilirubin molecules.

**Figure 14:**
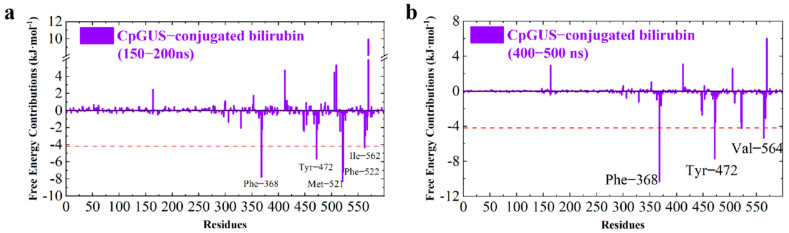
Per-residue free energy contribution of CpGUS and conjugated bilirubin calculated from the equilibrated 150–200 (**a**) and 400–500 ns (**b**) MD trajectory.

Per-residue free energy decomposition calculation was also performed for the CpGUS and conjugated bilirubin complex, and the results are shown in Figure 14:

As displayed in Figure 14 and listed in Table 6, several key residues in the CpGUS–conjugated bilirubin complex system exhibiting a binding affinity exceeding −4.18 kJ/mol during the 150–200 ns simulation period are identified, such as Phe368, Tyr472, Met521, Phe522, and Ile562. Their corresponding ∆*G_bind_* values are −7.80, −5.68, −8.32, −7.21, and −4.37 kJ/mol, respectively. During the 400–500 ns equilibrated stage, the ∆*G_bind_* values of three key residues (Phe368, Tyr472, and Val564) are found to be maintained above −4.18 kJ/mol, with their ∆*G_bind_* values of −10.33, −7.73, and −5.36 kJ/mol, respectively. These findings highlight the positive roles played by these three residues, which significantly contribute to the binding process of the CpGUS–conjugated bilirubin complex, especially for Phe368 and Tyr472, which are steadily present in both binding and catalysis at two different stages.

**Figure 15:**
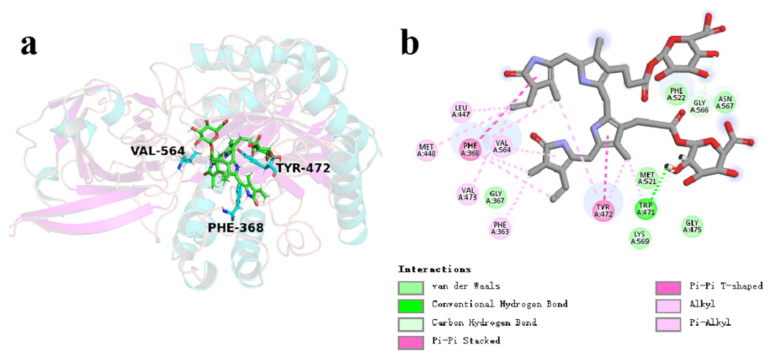
Key interaction analysis in the CpGUS–conjugated bilirubin system. (**a**) The primary conformation of CpGUS and conjugated bilirubin complex with key interactions at the active catalytic pocket under the equilibrated state. (**b**) Interaction mode diagram of CpGUS with the conjugated bilirubin.

As demonstrated in Figure 15, the conjugated bilirubin molecules steadily bind to the active catalytic cavity, which is comprised of non-polar residues, such as Phe363, Gly367, Leu447, Met448, Trp471, Val473, Gly475, Met521, Phe522, Val564, Gly566, Asn567, and Lys569, as well as polar residues Phe368 and Tyr472. In addition to the alkyl-π and π-π stacking interactions between the sidechains of Phe368, Tyr472, and Val564 and the benzene rings of conjugated bilirubin significantly enhanced the binding capacity of the CpGUS–conjugated bilirubin complex. Hydrophobic and hydrogen-bonding interactions also notably imposed important influences to the binding stability.

**Figure 16:**
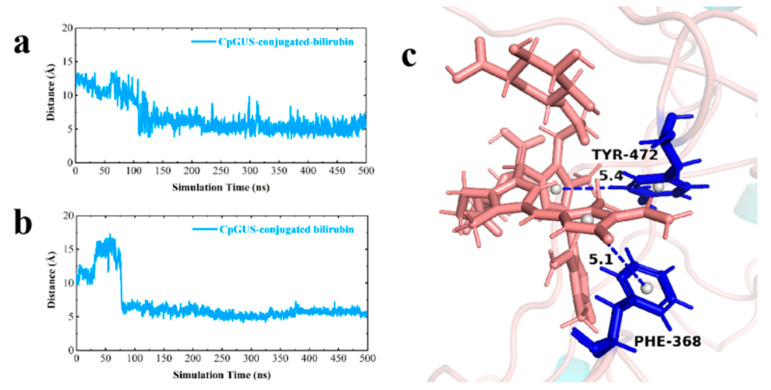
The time-dependent distance curves between key residues and conjugated bilirubin. (**a**) The distance curve of the π-π stacking interaction formed between the key residue Phe368 and the conjugated bilirubin; (**b**) The distance curve of the π-π stacking interaction formed between the key residue Tyr472 and the conjugated bilirubin; (**c**) The ribbon diagram of key interactions between key residues and conjugated bilirubin (unit: Å).

Despite a slight structural shift in the conjugated bilirubin during the initial MD process, stable π-π stack interaction between key residues Phe368 and Tyr472 and bilirubin were stabilized in the dominant conformation of CpGUS, and they significantly contributed to its binding dynamics and catalytic process (Figure 16a,b). Consequently, the substrate of bilirubin could be steadily anchored in the CpGUS active pocket and was effectively catalyzed by CpGUS to produce unconjugated bilirubin. The benzene ring distances between the key residues Phe368 and Tyr472 and the conjugated bilirubin were 5.1 and 5.4 Å (Figure 16c).

## 4. Discussion

β-Glucuronidase, a commonly employed glycosyl hydrolase, is utilized in the synthesis of various valuable compounds. The physicochemical characteristics of β-glucuronidase from diverse sources differ significantly. Notably, bacteria, such as *E. coli*, *Klebsiella* sp., *Bacteroides fragilis* [38], *Streptococcus* sp. [39], and *Staphylococcus* sp. [40], exhibit β-glucuronidase activity. Among these, *Escherichia coli* and *Clostridium perfringens* are prominent for their high production of the enzyme.

Although *E. coli* is recognized as a primary source, a review of the literature suggests that *Clostridium perfringens* exhibits greater enzyme activity compared to *E. coli* [32]. Consequently, this paper focuses on the enzymatic characteristics of β-glucuronidase CpGUS from *Clostridium perfringens*, examining the effects of pH, temperature, and metal ions on its activity and the efficiency of enzyme conversion. The results indicate that the optimal reaction temperature for CpGUS is 45 °C. CpGUS demonstrates good performance and high thermal stability within the temperature range of ≤45 °C, yet it has poor heat resistance. These findings are comparable to those of previously reported β-glucuronidase (GUS) enzymes, such as those from *Aspergillus terreus* and *Penicillium purpurogenum* Li-3 [41,42]. This similarity suggests that the majority of GUS enzymes are sensitive to temperature. Previous studies have revealed that the optimal pH values of GUS enzymes from different sources vary significantly. For instance, the optimal pH of GUS isolated from *Aspergillus niger* is 3.0 [42], the GUS obtained from baicalin and eubacteria exhibits maximum activity at pH 5.0 [43,44]; and the optimal pH of GUS from *Scutellaria baicalensis* callus ranges from 7.0 to 8.0 [45]. Most of the GUS enzymes investigated are active within the pH range of approximately 3.0–8.0 [43,44]. For example, the GUS of *P. purpurogenum* shows strong enzymatic activity in the pH range of 5–8 [41]. Baicalin-associated GUS displays high stability in the slightly acidic to neutral pH range (i.e., 4.0–7.0). *Aspergillus niger* β-GUS is stable in the pH range of 4.5–9.0 [46]. In the present study, the optimal pH of CpGUS was determined to be 5.0, and the activity of CpGUS is prone to be lost under overly acidic conditions over an extended period. Both previous findings and the results of this study suggest that most GUS enzymes are not highly sensitive to pH and exhibit stronger enzyme activity in acidic environments compared to alkaline environments. Under strongly acidic conditions or prolonged exposure to high temperatures, CpGUS shows reduced activity. It has been documented that Ca^2+^ and Mg^2+^ can enhance GUS activity [47]. Our results indicate that Fe^2+^ and Mg^2+^ enhance the activity of β-glucuronidase, whereas Zn^2+^, K^+^, Fe^3+^, Mn^2+^, Cu^2+^, and Na^^+^^ inhibit it, with Cu^2+^ and Fe^3+^ notably rendering CpGUS completely inactive. These insights assist in the enzymatic hydrolysis of pig bile to produce unconjugated bilirubin. The hydrolysis system should avoid Cu^2+^ and other disruptive metal ions, and operate in a low-temperature, mildly acidic environment to optimize reaction and storage conditions for CpGUS. Given that Fe^2+^ can convert to Fe^3+^, impairing CpGUS activity, Mg^2+^ was selected for the optimal conditions to conduct the hydrolysis of conjugated bilirubin in pig bile.

Whole-cell transformed pig bile was produced via high-density fermentation. The hydrolysis rate of conjugated bilirubin was 81.1%, and the yield and purity of unconjugated bilirubin were 76.8% and 98.2%, respectively. In this study, both the hydrolysis rate of conjugated bilirubin and the purity of unconjugated bilirubin exceeded those reported in previous studies [18,48]. However, the transformation of conjugated bilirubin in pig bile was not complete. This incompleteness may have resulted from a gradual decrease in CpGUS enzyme activity over time. Additionally, bile’s complex composition, including bile acids, cholesterol, and metal ions, may impair enzyme activity [49].

Although the reports on the crystal structures of GUS have gradually increased in recent years, the progress in the catalytic recognition mechanism of its substrates (including the glycosyl and aglycone moieties) has been relatively slow. The research group led by Wallace analyzed the complex crystal structure of EGUS and its inhibitor and concluded that the recognition of the glycosyl group of this inhibitor is achieved through multiple polar interactions formed with EGUS [24]. To investigate the hydrolysis mechanism of pNPG and conjugated bilirubin by CpGUS, the structure of the CpGUS protein model was predicted from its amino acid sequence. The best docked conformations of the CpGUS model with pNPG and conjugated bilirubin were adopted to conduct independent 500 ns molecular dynamics simulation. We found that Tyr472 is crucial for their binding and catalysis. Its sidechain could form the π-π stacking interactions with the benzene rings of the substrates. Strong hydrogen bonding and hydrophobic interactions maintained the binding stability of these two complexes. The total binding free energy (∆*G_bind_*) values were −65.05 ± 12.66 kJ/mol and −86.70 ± 17.18 kJ/mol, indicating high binding affinity and catalytic activity of CpGUS against these two substrates.

Currently, β-glucuronidase is primarily sourced from bacterial fermentation, which is hampered by long fermentation periods, low enzyme yields, and high costs, limiting industrial bilirubin production. Traditional chemical bilirubin production methods, involving high temperatures and strong alkalis, often result in the by-product biliverdin, leading to lower yields and significant environmental damage. Conversely, enzymatic conversion offers a more efficient and environmentally friendly alternative, showing considerable industrial potential. In this study, unconjugated bilirubin was successfully obtained from pig bile, with the hydrolysis rate, yield, and purity meeting industrial requirements. Future studies will focus on scaling up the fermentation and catalytic reaction volumes to facilitate industrial bilirubin production via enzymatic conversion. Additionally, combining chemical and biological techniques could further enhance bilirubin conversion efficiency. Bioinformatics may guide enzyme molecular modifications, and enhanced production and activity of CpGUS could be achieved through directed irrational design, semi-rational design [50], or rational design.

## Figures and Tables

**Table 1:** Kinetic parameters of CpGUS.

Concentration/mM	OD_405_	Enzyme Activity (U/μL)	1/[S] (1/mM)	1/V [1/(U/μL)]
0.4	0.674	86.13	2.500	0.0116
0.6	0.902	114.63	1.667	0.0087
0.8	1.003	127.25	1.250	0.0079
1.0	1.117	141.50	1.000	0.0071
1.2	1.220	154.38	0.833	0.0065
1.4	1.253	158.50	0.714	0.0063

**Table 2:** Binding free energy of the CpGUS–pNPG complex system calculated from 300 to 350 ns and 430 to 500 ns in the MD trajectory (unit: kJ/mol).

System	Time Scope/ns	ΔG*_vdw_*	ΔG*_ele_*	ΔG*_PB_*	ΔG*_SA_*	∆G*_bind_*
CpGUS–pNPG	300–350	−147.17 ± 14.36	−119.21 ± 9.53	216.63 ± 10.94	−15.30 ± 0.87	−65.05 ± 12.66
	430–500	−127.56 ± 14.62	−113.37 ± 18.78	194.37 ± 20.65	−14.72 ± 0.93	−61.28 ± 18.01

**Table 3:** Per-residue free energy decomposition (PFED) calculation for the CpGUS–pNPG complex during 300–350 ns and 430–500 ns MD simulation (unit: kJ/mol).

System	Time Scope/ns	Residue	ΔG*_MM_*	ΔG*_PB_*	ΔG*_SA_*	∆G*_bind_*
CpGUS–pNPG	300–350	Leu447	−6.83	2.90	−0.66	−4.60
		Asn466	−3.23	−3.87	−0.10	−7.21
		Tyr468	−11.04	3.29	−0.52	−8.29
		Tyr472	−17.33	8.93	−1.60	−9.99
	430–500	Phe368	−6.25	2.91	−0.68	−4.00
		Asn466	−3.20	−3.01	−0.11	−6.32
		Tyr468	−8.30	4.29	−0.47	−4.47
		Tyr472	−13.74	7.12	−1.29	−7.92

**Table 4:** Preparation of unconjugated bilirubin by whole-cell transformation of pig bile.

Serial Number	Concentration of Total Bilirubin in Primary Pig Bile (mg/mL)	Concentration of Total Bilirubin in Pig Bile After Hydrolysis Transformation (mg/mL)	Hydrolysis Conversion (%)	Quality of Unconjugated Bilirubin Extracted from 1 L of Pig Bile After Transformation (g)	Unconjugated Bilirubin Yield (%)
1	0.46	0.090	80.4	0.350	76.1
2	0.46	0.090	80.4	0.350	76.1
3	0.46	0.080	82.6	0.360	78.3
Mean value	0.46	0.087	81.1	0.353	76.8

Note: In the whole-cell transformation system using pig bile, *E. coli* BL21(DE3)/pET28a-CpGUS reached an OD_600_ of 10 at pH 5.0, with a temperature of 45 °C, a rotational speed of 200 rpm, and a reaction duration of 12 h.

**Table 5:** Binding free energy of the CpGUS and conjugated bilirubin complex system calculated from two different equilibrated stages (unit: kJ/mol).

System	Time Scope/ns	ΔG*_vdw_*	ΔG*_ele_*	ΔG*_PB_*	ΔG*_SA_*	∆G*_bind_*
CpGUS– Conjugated bilirubin	150–200	−221.44 ± 16.97	−41.68 ± 27.80	220.82 ± 52.65	−25.67 ± 2.02	−67.97 ± 36.72
	400–500	−200.12 ± 26.51	−35.36 ± 22.78	171.31 ± 26.11	−22.54 ± 2.15	−86.70 ± 17.18

**Table 6:** Per-residue free energy decomposition calculation for the CpGUS and conjugated bilirubin complexed system during the equilibrated 150–200 and 400–500 ns MD trajectory (Unit: kJ/mol).

System	Time Scope/ns	Residue	(ΔG*_MM_*)	(ΔG*_PB_*)	(ΔG*_SA_*)	(∆G*_bind_*)
CpGUS– Conjugated bilirubin	150–200	Phe368	−13.35	6.86	−1.32	−7.80
		Tyr472	−17.27	13.27	−1.66	−5.68
		Met521	−12.94	5.90	−1.27	−8.32
		Phe522	−9.36	3.61	−1.47	−7.21
		Ile562	−6.03	2.34	−0.65	−4.37
	400–500	Phe368	−15.87	7.43	−1.92	−10.33
		Tyr472	−23.16	17.64	−2.20	−7.73
		Val564	−6.59	2.51	−1.30	−5.36

## Data Availability

The original contributions presented in this study are included in the article. Further inquiries can be directed to the corresponding authors.
