# Correction: Wu et al. Heterologous Expression and Enzymatic Properties of β-Glucuronidase from *Clostridium perfringens* and Its Application in Bilirubin Transformation. *Microorganisms* 2025, *13*, 1043

**DOI:** 10.3390/microorganisms13102397

**Published:** 2025-10-20

**Authors:** Qianlin Wu, Qing Guo, Fo Yang, Mengru Li, Yumeng Zhu, Binpeng Xu, Lu Zhao, Shanshan Zhang, Youyu Xie, Feng Li, Xiaomin Wu, Dayong Xu

**Affiliations:** 1 Anhui Province Key Laboratory of Pollutant Sensitive Materials and Environmental Remediation, Huaibei Normal University, Huaibei 235000, China; 17334612971@163.com (Q.W.); yangfo0820@163.com (F.Y.); 18256188142@163.com (M.L.); 12211070750@chnu.edu.cn (Y.Z.); zss9042@163.com (S.Z.); xieyy@chnu.edu.cn (Y.X.); lifeng@chnu.edu.cn (F.L.); 2 School of Life Sciences, Huaibei Normal University, Huaibei 235000, China; 3 Anhui Chem-Bright Bioengineering Co., Ltd., Huaibei 235000, China; liangsui1980@163.com (Q.G.); xvb1996@sohu.com (B.X.); zhaolu@ahkebao.com (L.Z.); 4 Anhui Province Engineering Technology Research Center for Livestock by-Product Medical Intermediate Extraction, Huaibei 235000, China

## Error in Figure

In the original publication [1], there was a mistake in “Figure 13. The time-dependent root mean squared deviation (RMSD) curve of CpGUS and the conjugated bilirubin complexed system.” as published. Figure 13 was inadvertently duplicated from Figure 6. The corrected “Figure 13. The time-dependent root mean squared deviation (RMSD) curve of CpGUS and the conjugated bilirubin complexed system.” appears below. The authors state that the scientific conclusions are unaffected. This correction was approved by the Academic Editor. The original publication has also been updated.

## Figures and Tables

**Figure 13:**
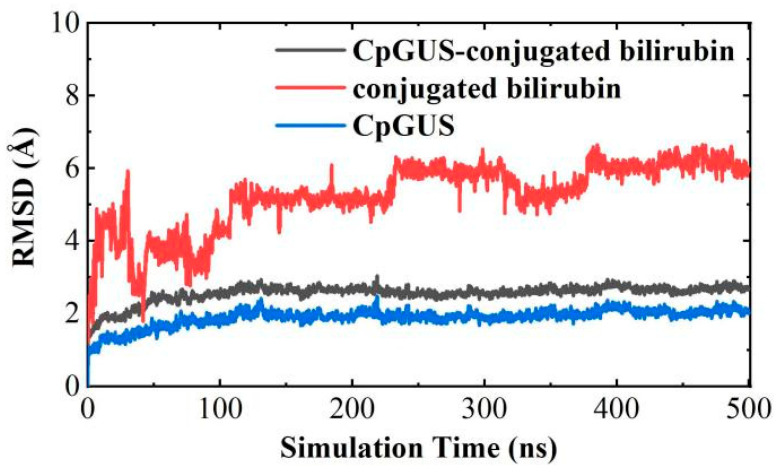
The time-dependent root mean squared deviation (RMSD) curve of CpGUS and the conjugated bilirubin complexed system.
